# Contrarian Majority Rule Model with External Oscillating Propaganda and Individual Inertias

**DOI:** 10.3390/e25101402

**Published:** 2023-09-30

**Authors:** Maria Cecilia Gimenez, Luis Reinaudi, Serge Galam, Federico Vazquez

**Affiliations:** 1 Instituto de Física Enrique Gaviola (IFEG-Conicet), Facultad de Matemática, Astronomía, Fśica y Computación (FaMAF, Universidad Nacional de Córdoba), Córdoba X5000HUA, Argentina; 2 Instituto de Investigaciones en Físico-Química de Córdoba (INFIQC, Conicet), Facultad de Ciencias Químicas (Universidad Nacional de Córdoba), Córdoba X5000HUA, Argentina; luis.reinaudi@unc.edu.ar; 3 CEVIPOF—Centre for Political Research, Sciences Po and CNRS, 1, Place Saint Thomas d’Aquin, 75007 Paris, France; serge.galam@sciencespo.fr; 4 Instituto de Cálculo, Facultad de Ciencias Exactas y Naturales, Universidad de Buenos Aires and Conicet, Intendente Guiraldes 2160, Cero + Infinito, Buenos Aires C1428EGA, Argentina

**Keywords:** opinion dynamics, majority rule model, noise, periodic field, stochastic resonance

## Abstract

We study the Galam majority rule dynamics with contrarian behavior and an oscillating external propaganda in a population of agents that can adopt one of two possible opinions. In an iteration step, a random agent interacts with three other random agents and takes the majority opinion among the agents with probability p(t) (majority behavior) or the opposite opinion with probability 1−p(t) (contrarian behavior). The probability of following the majority rule p(t) varies with the temperature *T* and is coupled to a time-dependent oscillating field that mimics a mass media propaganda, in a way that agents are more likely to adopt the majority opinion when it is aligned with the sign of the field. We investigate the dynamics of this model on a complete graph and find various regimes as *T* is varied. A transition temperature $T_{c}$ separates a bimodal oscillatory regime for $T<T_{c}$, where the population’s mean opinion *m* oscillates around a positive or a negative value from a unimodal oscillatory regime for $T>T_{c}$ in which *m* oscillates around zero. These regimes are characterized by the distribution of residence times that exhibit a unique peak for a resonance temperature $T^{*}$, where the response of the system is maximum. An insight into these results is given by a mean-field approach, which also shows that $T^{*}$ and $T_{c}$ are closely related.

## 1. Introduction

In recent decades, statistical physics has expanded its scope to venture into the field of sociology, giving rise to a discipline called sociophysics [1,2,3,4,5,6,7,8,9,10]. A commonly studied phenomenon is the dynamics of opinion formation, by means of simple mathematical models. In these models, individuals are called agents, and each of them is characterized by the value of a variable that represents its opinion on a particular topic—such as the intention to vote for a candidate on a ballot—which, for simplicity, can take one of two possible values (+1 or −1). The opinion of each agent can change after interacting with other agents following simple rules. One of the most implemented interaction rules is that introduced in a model by Galam [11] and extensively studied later on [6,12,13,14], to which we refer as the Galam Majority Model (GMM), in which all agents in a group chosen at random adopt the opinion of the majority in that group. This local dynamic drives a steady increase in the initial global majority opinion (provided the system’s symmetry is not broken by ties for even-size groups), which eventually ends with a consensus, i.e., an absorbing state where all agents share the same opinion. Multiple extensions of the GMM have been studied in the literature, including the possibility of contrarian behavior, that is, all members of a chosen group taking the minority opinion [7]. This work studied the effects of introducing a fixed fraction *a* of contrarian agents to the original GMM, where it was found that, instead of a frozen consensus, as in the model with no contrarians, the system reaches an ordered stationary state for $a<a_{c}$ and a disordered stationary state for $a>a_{c}$. The transition value $a_{c}$ separates an ordered phase, where a large majority of agents hold the same opinion, from a disordered phase, in which both opinions are equally represented in the population.

Many other opinion formation models with contrarians were also studied in [15,16,17,18,19,20,21,22,23,24,25,26,27,28,29,30,31,32,33,34]. In particular, the effects of contrarian behavior were also investigated in the voter model (VM) for opinion formation [24], where agents interact in pairs and one adopts the opinion of the other, with a probability of 1−P (imitation), or the opposite opinion, with a probability of P (contrarian). It was shown that the model displays a transition from order to disorder when the probability of having a contrarian behavior overcomes the threshold $P_{c}={\left(N+1\right)}^{-1}$ in a system of *N* agents. The contrarian voter model [24] was recently studied under the presence of a mass media propaganda that influences agents’ decisions [34]. The propaganda was implemented in the form of an external oscillating field that tends to align agents’ opinions in the direction of the field. A stochastic resonance (SR) phenomena within an oscillatory regime was found, that is, there is an optimal level of noise for which the population effectively responds to the modulation induced by the external field [35,36].

In order to expand our knowledge on the combined effects of contrarians and mass media propaganda on opinion models, we study in this article the GMM with contrarian behavior under the presence of an external oscillating field. Each agent in the population can either follow a majority rule that increases similarity with its neighbors or behave as a contrarian by adopting the opposite opinion, with respective probabilities p(t) and 1−p(t). The majority probability p(t) varies periodically in time according to an external field or propaganda, based on a mathematical form introduced in [21,22] for the Sznajd model and implemented in [34,37] for the VM, so that agents tend to follow the majority when it is aligned with the field. Although we understand that this model is relatively far from being adequate to describe real social systems where mass media does not necessarily change sign periodically, it provides analytical insight on features that could be actually relevant in the real word. We also notice that, unlike models where a predetermined group of agents always behaves as a contrarian (adopt the minority opinion) and the rest of agents always behave as a follower (adopt the majority opinion), like in [7], in our model each agent can behave as both, as a contrarian or as a follower at each iteration step of the dynamics, with a frequency of choosing the contrarian behavior given by 1−p(t). However, we note that these two models lead to identical mean-field (MF) equations for the system’s evolution in an all-to-all interaction setup. By exploring the dynamics of the GMM under the influence of an oscillating external field and the presence of contrarians, we aim to gain deeper insights into the manifestation of the SR phenomenon in opinion dynamics models. We show that this model exhibits unimodal and bimodal oscillatory regimes, as well as an SR that is observed close to the transition between the two regimes.

It is worth mentioning that the GMM studied in this article belongs to a class of “non-linear” models, while the VM with contrarians described above and studied in [34] belongs to a completely different class. That is, in the VM an agent switches opinion (s→−s, s=+1,−1) with a probability equal to the fraction of neighbors that hold the opposite opinion $\sigma_{-s}$ (linear in $\sigma_{-s}$), while in the GMM the switch happens with a probability that is proportional to a combination of $\sigma_{-s}^{2}$ and $\sigma_{-s}^{3}$, i.e., non-linear in $\sigma_{-s}$. Then, the equation that governs the time evolution of the fraction of agents in a given opinion can be written as a Ginzburg–Landau equation with an associated potential that has two symmetric wells in the GMM, as in the Ising model for ferromagnetism, while in the VM the potential is zero. A main consequence of this difference in the potential is in the type of domain coarsening observed in spatial systems: while in the GMM the coarsening is driven by curvature, in the VM it is without surface tension [38] (driven by noise). Another consequence is that, in the version of these models with contrarians, the order–disorder transition in the thermodynamic limit (N→∞) takes place at a finite fraction of contrarian agents $a_{c}>0$ in the GMM, while in the VM the transition happens at a vanishing contrarian probability ($P_{c}\to 0$).

We also need to mention that the SR effect has also been observed in other opinion models. For instance, in [21,22] the authors found SR in a variation of the Sznajd model with stochastic driving and a periodic signal. The work in [14] analyzed a majority rule dynamics under the action of noise and an external modulation, and found an SR that depends on the randomness of the small-world network. There are also other works [39,40,41,42,43] that explored the combined effects of stochastic driving and an external signal on a majority rule dynamics. However, none of these works have incorporated a contrarian behavior in the dynamics.

The rest of this article is organized as follows. We introduce the model in Section 2. In Section 3, we present numerical simulation results for the evolution of the system and the behavior of different magnitudes that characterize the SR phenomena. In Section 4, we develop an MF approach that gives an insight into the system’s evolution and the relation between the SR and the transition between different regimes. Finally, in Section 5 we summarize our findings and discuss the results.

## 2. The Model

We consider a population of *N* interacting agents where a given agent *i* (i=1,…,N) can hold one of two possible opinion states, $s_{i}=+1,-1$. We denote by $\sigma_{+}\left(t\right)$ and $\sigma_{-}\left(t\right)$ the fraction of nodes with respective states +1 and −1 at time *t*, such that $\sigma_{+}\left(t\right)+\sigma_{-}\left(t\right)=1$ for all t≥0. In a time step Δt=1/N of the dynamics, we follow the basic GMM using groups of size three to update individual opinions. However, here, for our purpose of investigating the effects of propaganda at the level of a single individual, we implement the rule in a different setting, where only one individual can update its opinion state in an iteration step. Instead of selecting three agents randomly to update all of them at once, we pick one agent *i* with state $s_{i}$ and a group of three other different agents j,k,l (i≠j≠k≠l), all randomly chosen. In the N≫1 limit, their respective states are $\left(s_{j},s_{k},s_{l}\right)$ with probability $\sigma_{s_{j}}\sigma_{s_{k}}\sigma_{s_{l}}$. A majority of + choices is thus obtained for the configurations (+,+,+), (+,+,−), (+,−,+) and (−,+,+), yielding an overall probability

(1) $$P_{+}\equiv \sigma_{+}^{3}+3\sigma_{+}^{2}\sigma_{-}.$$

Similarly, a majority of − occurs for (−,−,−), (+,−,−), (−,+,−) and (−,−,+), with the overall probability

(2) $$P_{-}\equiv \sigma_{-}^{3}+3\sigma_{-}^{2}\sigma_{+}.$$

Then, agent *i* updates its state in two steps. (i) First, the update follows the basic GMM, where agent *i* simply adopts the majority state of the group of the three agents j,k and *l*. We thus have $s_{i}\to s_{i}=+1$ with probability $P_{+}$, or $s_{i}\to s_{i}=-1$ with probability $P_{-}=1-P_{+}$. (ii) Second, agent *i* can either preserve this majority state ($s_{i}\to s_{i}$) with probability $p_{s_{i}}$, or change to the opposite (minority) state ($s_{i}\to -s_{i}$) with the complementary probability $1-p_{s_{i}}$, where $p_{s_{i}}$ is defined below. The implication of this second step is that each agent can behave as a “contrarian” by adopting the state opposed to the majority (minority state) with probability $1-p_{s_{i}}$, or as a “majority follower” with probability $p_{s_{i}}$. Thus, there is no fixed fraction of contrarian agents, as in [7].

At this point, we introduce the effect of an external field *H* on agent *i* in state $s_{i}$ within a Boltzmann scheme by assuming that the probability $p_{s_{i}}$ to preserve the majority state is larger when $s_{i}$ is aligned with *H* [i.e., $\mathrm{sign}\left(s_{i}\right)=\mathrm{sign}\left(H\right)$],

(3) $$p_{s_{i},H}=\frac{e^{\left[s_{i}H\right]/T}}{e^{\left[s_{i}H\right]/T}+e^{-\left[s_{i}H\right]/T}}\;,$$

where T≥0 is a parameter that plays the role of a *social temperature* analogous to the contrarian feature of the GMM. The related probability to oppose the field is $1-p_{s_{i},H}$. We assume that *H* is an oscillating periodic field $H\left(t\right)=H_{0}\sin\left(\omega t\right)$ with amplitude $H_{0}$ ($0\leq H_{0}\leq 1$), frequency ω=2π/τ and period τ, which represents an external propaganda. Thus, according to Equation (3), agents are more likely to keep the opinion that is aligned with the propaganda. In addition to the external field, we introduce an individual “inertia” parameter *I*, which provides an agent with a weight to preserve its current state against a field favoring the opposite state. It is a self-interaction $-Is_{i}s_{i}$ which modifies Equation (3) as

(4) $$p_{s_{i},a,H}=\frac{e^{\left[Is_{i}+H\right]s_{i}/T}}{e^{\left[Is_{i}+H\right]s_{i}/T}+e^{-\left[Is_{i}+H\right]s_{i}/T}}\;,$$

which can be rewritten as

(5) $$p_{s_{i},1,H}=\frac{e^{\left[1+s_{i}H\right]/T}}{e^{\left[1+s_{i}H\right]/T}+e^{-\left[1+s_{i}H\right]/T}}\;,$$

where I,H and *T* have been rescaled as $1,\frac{H}{I}$ and $\frac{T}{I}$ using $s_{i}^{2}=1$.

At this stage, we combine the GMM with the inertia and field effects by taking

(6) $$p_{s_{i}}\left(t\right)=\frac{e^{\left[1+s_{i}H\left(t\right)\right]/T}}{e^{\left[1+s_{i}H\left(t\right)\right]/T}+e^{-\left[1+s_{i}H\left(t\right)\right]/T}}$$

for the probability of agent *i* to keep the majority state $s_{i}$, and $1-p_{s_{i}}\left(t\right)$ for the probability to adopt the opposite (minority) state $-s_{i}$, which can be interpreted as a noise. Finally, combining Equations (1), (2) and (6), the probability $P_{+}$ for a randomly selected agent *i* to adopt the state + in a single time step Δt is given by

(7) $$\begin{array}{c} P_{+}=\left(\sigma_{+}^{3}+3\sigma_{+}^{2}\sigma_{-}\right)\frac{e^{\left[1+H(t)\right]/T}}{e^{\left[1+H(t)\right]/T}+e^{-\left[1+H(t)\right]/T}}+\left(\sigma_{-}^{3}+3\sigma_{-}^{2}\sigma_{+}\right)\frac{e^{-\left[1-H(t)\right]/T}}{e^{\left[1-H(t)\right]/T}+e^{-\left[1-H(t)\right]/T}}\;, \end{array}$$

where the first term comes from following a local majority + among the three selected agents, which happens with probability $P_{+}p_{+}\left(t\right)$, while the second term corresponds to opposing the state − in case of a majority of − among the three selected agents, which happens with probability $P_{-}\left[1-p_{-}\left(t\right)\right]$. Analogously, the state − is selected with probability $P_{-}\equiv 1-P_{+}$.

As noted above, in a single iteration only one agent (the “focal agent” *i*) updates its state, unlike in the original Galam’s model where all three agents in the chosen group update their states at once [11]. However, as these two settings use the same majority rule and they differ only in the number of agents updated in an iteration, they turn out to be equivalent at the MF level, i.e., in an all-to-all interaction setup. That is, they both have the same rate equations for the evolution of macroscopic quantities (see Section 4). Therefore, the model introduced in this section is the same as the original Galam’s model, with the novelty of the addition of an external oscillating field that is coupled to agents’ opinions, which extends the parameter space of Galam’s model and leads to new phenomena, as we shall see.

Equation (6) shows that individuals are more prone to adopt the opinion of the majority when it is aligned with the propaganda. In addition, $p_{+}$ and $p_{-}$ approach the value one as T→0, which makes this case equivalent to the original GMM, with neither contrarians nor an external field. In the opposite limit T→∞, $p_{+}$ and $p_{-}$ approach the value 1/2, which corresponds to the pure noise case where agents take one of the two opinions at random, independent of the field.

In the Glossary, we display a table with a list of the symbols of variables and their names that we use throughout the text.

## 3. Numerical Results

### 3.1. Evolution of the Magnetization

**Figure 1:**
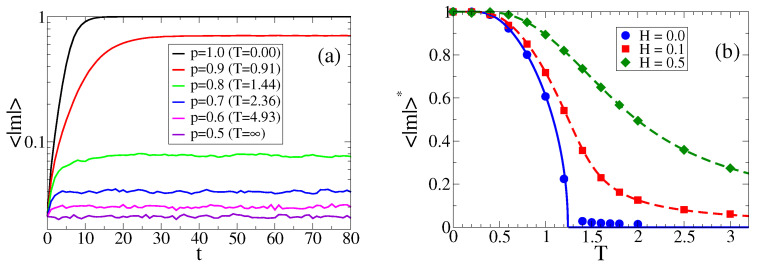
(**a**) Time evolution of the average value of the absolute magnetization |m| in a population of $N=10^{3}$ agents, zero field H=0 and various values of majority probability $p={\left(1+e^{-2/T}\right)}^{-1}$, as indicated in the legend. (**b**) Stationary value of 〈|m|〉 vs. temperature *T* for constant fields H=0.0 (circles), H=0.1 (squares) and H=0.5 (diamonds). The solid line is the analytical expression from Equation (11), while the dashed lines are the numerical integration of Equation (8). The averages were performed over $10^{3}$ independent realizations starting from a symmetric condition $m_{0}=0$.

We start by studying the time evolution of the mean opinion of the population, or magnetization, defined as $m\left(t\right)\equiv \frac{1}{N}\sum_{i=1}^{N}s_{i}\left(t\right)$, for the simplest case of zero field H=0, which corresponds to the contrarian GMM with symmetric majority probabilities $p_{+}=p_{-}=p={\left(1+e^{-2/T}\right)}^{-1}$. We run several independent realizations of the dynamics where, initially, each agent adopts state +1 or −1 with respective probabilities $\sigma_{+}\left(0\right)$ and $\sigma_{-}\left(0\right)$, leading to an initial average magnetization $m\left(0\right)=\sigma_{+}\left(0\right)-\sigma_{-}\left(0\right)$. Due to the symmetry of the system, the evolution of the average value of *m* over many realizations starting from m(0)=0 gives 〈m〉(t)≃0 for all t≥0, which does not describe the correct behavior of the system. Instead, we look at the evolution of the absolute value of the magnetization, |m|, as we show in Figure 1a, for various values of *p*. In Figure 1b we show in circles the stationary value of 〈|m|〉 (${\langle |m|\rangle }^{*}$) as a function of *T* for H=0. We observe that, as *T* increases, the system displays a transition from an ordered state, (|m|>0) for $T<T_{c}^{0}$, to a disordered state, (|m|≃0) for $T>T_{c}^{0}$, where $T_{c}^{0}$ is a transition temperature. This order–disorder transition, reminiscent of the GMM with a fixed fraction of contrarian agents [7], is induced by the presence of a contrarian behavior that acts as a source of external noise, preventing the system from reaching full consensus. When the noise amplitude, controlled by *T*, overcomes a threshold value $T_{c}^{0}$, the system reaches complete disorder. In Section 4, we develop an MF approach that allows one to estimate the transition temperature as $T_{c}^{0}≃1.24$. When the field is turned on, these results change completely. In the case that the field remains constant in time (constant propaganda H = const), the symmetry of the system is broken in direction of *H*, increasing the stationary value of 〈|m|〉 as compared with the H=0 case. This effect can be seen in Figure 1b, where we see that ${\langle |m|\rangle }^{*}$ increases monotonically with *H*. Additionally, the order–disorder transition disappears for H>0 (see the H=0.1 and H=0.5 curves).

**Figure 2:**
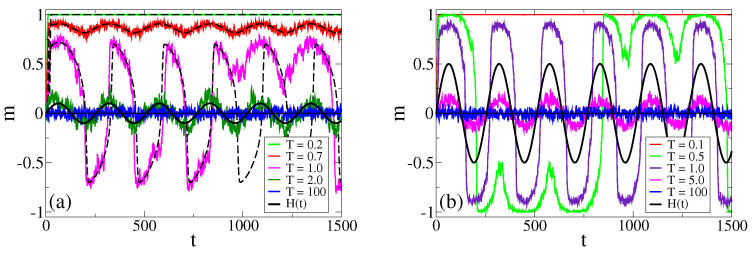
Time evolution of the magnetization *m* in a single realization for a population of N=1024 agents under the influence of an oscillating field with period τ=256 and amplitudes $H_{0}=0.1$ and 0.5, panels (**a**) and (**b**), respectively, and the temperatures indicated in the legends. Solid lines correspond to MC simulations, while dashed lines in panel (**a**) represent the numerical integration of Equation (8).

If we now let the field oscillate in time, a series of different regimes emerge. In Figure 2, we show the evolution of *m* in a single realization under the effects of an oscillating field, for three different amplitudes $H_{0}$, period τ=256 and various temperatures. For the $H_{0}=0.1$ and $H_{0}=0.5$ cases [panels (a,b)], we can see that for low temperatures *m* oscillates around a positive value or negative value, and that oscillations vanish for small enough *T* values, where *m* stays in a value close to 1.0 (consensus), as we can see for T=0.2 and T=0.1 in panels (a) and (b), respectively. The center of oscillations can jump from positive to negative values and vice versa (bimodal regime), as we can see in panel (b) for T=0.5. Above a given temperature threshold, $T_{c}≃1.0$ for $H_{0}=0.1$ [panel (a)] and $T_{c}≃0.5$ for $H_{0}=0.5$ [panel (b)], the magnetization oscillates around m=0 (unimodal regime). This behavior is reminiscent of the ordered and disordered phases in the model without a field [Figure 1b], although the transition temperature $T_{c}^{0}≃1.24$ for H=0 is quite different from that of the model with an oscillating field. An insight into this behavior shall be given in Section 4. For $H_{0}=1.0$ [panel (c)], oscillations are centered at m=0 even for small *T*, and, thus, the bimodal regime is not observed. Finally, at very large temperatures, the high levels of noise lead to a purely stochastic dynamics where agents adopt an opinion at random, and, thus, *m* fluctuates around zero.

### 3.2. Residence Times

**Figure 3:**
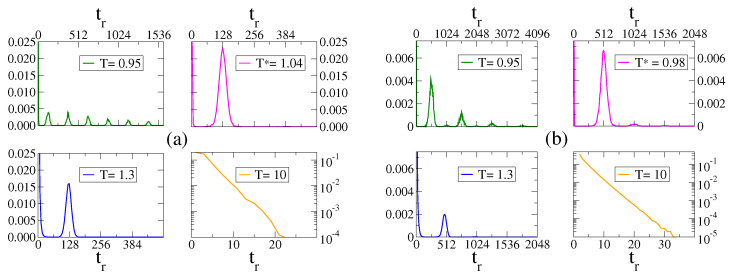
Normalized histograms of the residence time $t_{r}$ in a system of N=1025 agents under a field of amplitude $H_{0}=0.1$, period τ=256 (**a**) and τ=1024 (**b**), and the temperatures indicated in the legends. The bottom-right panels are on a linear-log scale.

In order to characterize the different regimes described in the last section, we study here the residence time $t_{r}$, defined as the time interval between two consecutive changes of the sign of *m*, i.e., when *m* crosses the center value m=0. In a single realization, *m* can change sign multiple times depending on the parameter values, leading to a distribution of the residence time that is particular of each regime. Results are shown in Figure 3 for N=1025, $H_{0}=0.1$, τ=256 [panel (a)] and τ=1024 [panel (b)]. In the unimodal regime, *m* follows the oscillations of H(t) around zero, and, thus, *m* tends to change sign when *H* does, every time the interval is τ/2. Therefore, the residence time distribution (RTD) is peaked at $t_{r}≃\tau /2$, as shown in panel (a) for temperatures T=1.04 and T=1.3, and in panel (b) for T=0.98 and T=1.3. In the bimodal regime, the RTD exhibits multiple peaks at $t_{r}=\left(n+1/2\right)\tau$ (n=0,1,2,…) (see panels for T=0.95). Here, *m* tends to perform oscillations around a positive (negative) value until it changes to negative (positive) oscillations, and back to positive (negative) oscillations again, as we observe in Figure 2b for T=0.5. These changes are more likely to happen when *H* changes sign, in the first attempt at time t=τ/2, in the second attempt one period later (at t=3τ/2), in the third attempt at t=5τ/2 and so on, leading to the different peaks in the RTD. Finally, for very large *T* the RTD shows an exponential decay due to the stochastic fluctuations of *m* around zero (panels for T=10).

### 3.3. Stochastic Resonance

**Figure 4:**
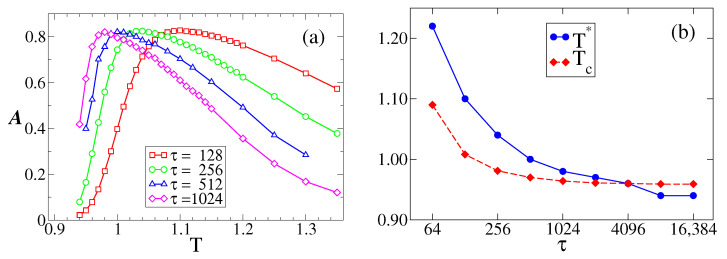
(**a**) Response A as a function of the temperature *T* for a field of amplitude $H_{0}=0.1$ and period τ indicated in the legend. (**b**) Resonance temperature $T^{*}$ [maximum of A vs. *T* curves from (**a**)] and transition temperature $T_{c}$ vs. period τ.

The patterns of the RTD shown in Section 3.2 can be employed to quantify the phenomena of stochastic resonance, as it was conducted in related systems [14,35]. The sensitivity or response of the system to the external oscillating field can be measured by the area A under the first peak around τ/2 in the RTD histogram. It is expected that A reaches a maximum at the resonance temperature $T^{*}$, when *m* resonates with the field *H*. This method to quantify the resonance is an alternative to the study of the signal-to-noise ratio [21,22,34]. Figure 4a shows the response A vs. *T* for a field of amplitude $H_{0}=0.1$. Each curve corresponds to a different period τ. We observe that A reaches a maximum value at a temperature $T^{*}$ that depends on τ. The RTD for the resonance temperatures $T^{*}=1.04$ and $T^{*}=0.98$ for periods τ=256 and τ=1024, respectively, are shown in the top-right panels of Figure 3a,b, where we see the existence of a well-defined peak centered at $t_{r}=\tau /2$. For larger temperatures (see T=1.3), there is also a peak at τ/2, although lower than that for $T^{*}$, and the RTD exhibits another pronounced peak near $t_{r}=0$, corresponding to the short crossings of m(t) that become more frequent as *T* increases (larger fluctuations in *m*).

## 4. Mean-Field Approach

In this section, we analyze the behavior of the model within an MF approach by deriving a rate equation for the evolution of *m* that corresponds to the dynamics introduced in Section 2. Let us write the fractions of + and − agents in terms of the magnetization *m*, $\sigma_{+}=\left(1+m\right)/2$ and $\sigma_{-}=\left(1-m\right)/2$. As we described in Section 2, in a time step Δt=1/N, a random agent *i* with state $s_{i}=-1$ is chosen with probability $\sigma_{-}$, and then adopts the state + ($s_{i}=-1\to s_{i}=+1$ flip) with probability $P_{+}=P_{+}p_{+}+P_{-}\left(1-p_{-}\right)$, which corresponds to adopt either the majority state + of a selected + majority, or the minority state + of a selected − majority, where $P_{+}$ and $P_{-}$ are given by Equations (1) and (2), respectively. This flip −1→+1 leads to an overall change Δm=2/N in *m*. Conversely, with probability $\sigma_{+}$, the chosen agent *i* has state +1, and flips to −1 ($s_{i}=+1\to s_{i}=-1$ flip) with probability $P_{-}=P_{-}p_{-}+P_{+}\left(1-p_{+}\right)$, leading to a change Δm=−2/N. Assembling these factors, the mean change of *m* in a time step can be written as

$$\begin{array}{ccc} & & \frac{dm}{dt}=\frac{1}{1/N}\left[\sigma_{-}P_{+}\left(\frac{2}{N}\right)-\sigma_{+}P_{-}\left(\frac{2}{N}\right)\right], \end{array}$$

which becomes, in the N→∞ limit, the rate equation

(8) $$\begin{array}{c} \frac{dm}{dt}=\frac{1}{2}m\left(m^{2}-5\right)+\frac{1}{2}p_{+}{\left(1+m\right)}^{2}\left(2-m\right)-\frac{1}{2}p_{-}{\left(1-m\right)}^{2}\left(2+m\right), \end{array}$$

after replacing the expressions for $P_{+}$ and $P_{-}$ and doing some algebra. Here,

(9) $$p_{+}\left(t\right)=\frac{e^{\left[1+H(t)\right]/T}}{e^{\left[1+H(t)\right]/T}+e^{-\left[1+H(t)\right]/T}}\;\;\;\mathrm{and}\;\;\;p_{-}\left(t\right)=\frac{e^{\left[1-H(t)\right]/T}}{e^{\left[1-H(t)\right]/T}+e^{-\left[1-H(t)\right]/T}}$$

are the probabilities of adopting the state +1 and −1 of a majority, respectively, as defined in Equation (6).

We note that if we consider the original Galam’s model where, in an iteration step, a group of three agents are chosen at random and all adopt the majority state, we can derive a rate equation for *m* that has the same form as Equation (8), with a prefactor of three multiplying the right-hand-side of the equation. Therefore, both models turn out to be equivalent in MF; they have the same stationary states and the same behavior. They only differ by a factor of three in the time scale associated with the relaxation to the steady state.

For the zero field case, ($H_{0}=0$) is $p_{+}=p_{-}=p={\left(1+e^{-2/T}\right)}^{-1}$, and, thus, Equation (8) is reduced to the simple equation

(10) $$\begin{array}{c} \frac{dm}{dt}=\frac{1}{2}m\left[6p-5-\left(2p-1\right)m^{2}\right]. \end{array}$$

Equation (10) has three fixed points corresponding to the possible stationary states of the agent-based model. The fixed point $m_{0}^{*}=0$ is stable for p<5/6 and corresponds to a disordered active state with equal fractions of + and − agents ($\sigma_{+}=\sigma_{-}=1/2$), whereas the two fixed points

(11) $$m_{\pm }^{*}=\pm \sqrt{\frac{6p-5}{2p-1}}$$

are stable for p>5/6, and they represent asymmetric active states of coexistence of + and − agents, with stationary fractions $\sigma_{+}^{*}=\left(1+m_{+}^{*}\right)/2>\sigma_{-}^{*}=\left(1-m_{+}^{*}\right)/2$ and $\sigma_{+}^{*}=\left(1+m_{-}^{*}\right)/2<\sigma_{-}^{*}=\left(1-m_{-}^{*}\right)/2$. The stable fixed points are plotted with a solid line in Figure 1b, where we observe a good agreement with MC simulation results (solid circles). Equation (11) shows the existence of a transition from order to disorder as *T* overcomes the value $T_{c}^{0}=2/\ln\left(5\right)≃1.24$ ($p_{c}^{0}=5/6$), as we already mentioned in Section 3.1. Notice that the probability of behaving as a contrarian $1-p_{c}^{0}=1/6$ is identical to the critical proportion of contrarians $a_{c}=1/6$ obtained in the GMM for groups of size three [7]. Given that Equation (10) can be rewritten as a Ginzburg–Landau equation with an associated double-well potential with two minima at $m_{\pm }^{*}$, we expect a bistable regime for $T<T_{c}$, where, in a single realization, *m* jumps between $m_{+}^{*}$ and $m_{-}^{*}$.

For a field that is constant in time (H=const≠0), the fixed points of Equation (8) are given by the roots of a cubic polynomial, and m=0 is no longer a root. Only one root is real, and corresponds to the stationary state of the agent-based model. As the analytical expression for the real root is large and not very useful, we integrated Equation (8) numerically to find the stationary value $m^{*}$, which we plot with a dashed line in Figure 1b for H=0.1 and 0.5. We observe a good agreement with MC simulations (symbols). A positive field H>0 breaks the symmetry in favor of the + state, given that $p_{+}>p_{-}$, leading to a positive stationary value $m^{*}>0$ that increases monotonically with *H*.

**Figure 5:**
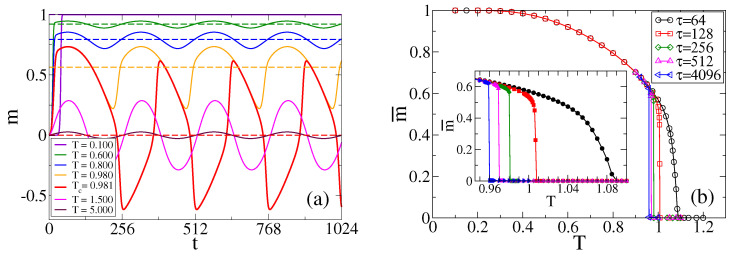
(**a**) Time evolution of the magnetization *m* from Equation (8) for a field of amplitude $H_{0}=0.1$, period τ=256 and the temperatures indicated in the legend. Horizontal dashed lines represent the time average value of *m*, $\bar{m}$, in the interval t∈(0,1000τ). (**b**) Time average of the magnetization, $\bar{m}$, vs. temperature *T* for the field’s periods indicated in the legend. The inset shows a closer look around the transition values $T_{c}$.

For an oscillating field H(t), we have that $p_{+}\left(t\right)$ and $p_{-}\left(t\right)$ oscillate in time according to H(t), which in turn leads to oscillations in m(t). In order to explore, within the MF approach, the behavior of *m* in the different regimes described in Section 3.1, we plot in Figure 5a the evolution of *m* obtained from the numerical integration of Equation (8) for $H_{0}=0.1$, τ=256 and various temperatures. For low temperatures, we see that *m* oscillates around a positive value (it could also be a negative value for other initial conditions), but when the temperature is increased beyond a threshold value, oscillations turn to be around m=0. At first sight, this transition that happens in the oscillatory regime of *m*, already reported in Section 3.1 from MC simulations, appears to be quite sharp, where the center of oscillations seems to jump from a large value to zero after a small increment of *T*. To better characterize the transition, we plot in Figure 5b the temporal average of *m* from t=0 to t=1000τ, called $\bar{m}$, as a function of *T* and for several periods τ. The value of $\bar{m}$ can be seen as an order parameter, which takes a positive or negative value in the bimodal regime and a value close to zero in the unimodal regime. We can see that $\bar{m}$ decreases continuously with *T* for low τ (see curve for τ=64), and that the transition becomes more abrupt as τ increases (see curves for τ≥256). The inset shows a more detailed view of the transition in the value of $\bar{m}$.

In Figure 2a, we compare the evolution of *m* obtained from the MF approach (dashed lines) with that from MC simulations, for $H_{0}=0.1$, τ=256 and various temperatures. We observe a good agreement with single realizations of the dynamics, except for the temperature T=1.0, which is close to the transition value $T_{c}≃0.981$, estimated from Figure 5b as the point where $\bar{m}$ becomes zero. This discrepancy is due to the fact that the MF approach cannot reproduce the random jumps of $\bar{m}$ from the value $\bar{m}≃0.564$ in the bimodal regime to $\bar{m}≃0$ in the unimodal regime. These jumps are induced by finite-size fluctuations, and are more frequent when the control parameter *T* is close to the transition point $T_{c}$.

An insight into the behavior of the resonance temperature $T^{*}$ with the period τ can be obtained from the MF approach, assuming that the response A reaches a maximum value at a temperature similar to the transition point $T_{c}$, that is, we expect, $T^{*}≃T_{c}$. This is because, in the bimodal regime $T<T_{c}$, the magnetization *m* oscillates around a positive or a negative value and eventually crosses m=0 around times t=τ/2, 3τ/2, etc., via finite-size fluctuations, leading to multiple peaks in the residence time distribution. Then, at $T=T_{c}$, oscillations start to be centered at $\bar{m}=0$, and, thus, we expect that the RTD shows a single peak at τ/2. By increasing *T* beyond $T_{c}$, we expect that the height of the peak for $T=T_{c}$ is reduced by the presence of a higher noise that induces another maximum of the RTD at t=0, as explained in Section 3.2, leading to a smaller A. Therefore, we expect that A is at its maximum at $T≃T_{c}$. Figure 4b shows in diamonds the value of $T_{c}$ obtained from Figure 5b for various periods τ. We see that $T_{c}$ decreases with τ, as it happens with $T^{*}$ (circles), although discrepancies between $T_{c}$ and $T^{*}$ increase as τ decreases.

## 5. Summary and Discussion

In this article, we studied the dynamics of the binary-state majority rule model introduced by Galam for opinion formation under the presence of an external propaganda and contrarian behavior. When an agent has to update its opinion, it can either follow the majority opinion among three random neighbors, similarly to the original GMM, or adopt the opposite (contrary) opinion, i.e., the minority opinion. The probability to adopt the majority opinion $p_{\pm }\left(t\right)$ is coupled to an external field that oscillates periodically in time (propaganda), in a way that agents are more likely to adopt the majority opinion when it is align with the field. This rule tries to reproduce a reinforcing mechanism by which individuals have a tendency to follow the majority opinion when it is in line with mass media propaganda. Additionally, the majority probability $p_{\pm }$ depends on a parameter *T* (temperature), which acts as an external source of noise, in such a way that by increasing *T* from zero the system goes from following the majority opinion only ($p_{\pm }=1$ for T=0) to adopting a random opinion for large temperatures ($p_{\pm }=0.5$ for T≫1).

We explored the model in a complete graph (all-to-all interactions) and found different phenomena associated with different regimes as *T* is varied. For *T* below a threshold value $T_{c}$, the system is in a bimodal regime, where the mean opinion *m* oscillates in time around a positive or negative value, ${\bar{m}}_{\pm }$, and performs jumps between ${\bar{m}}_{+}$ and ${\bar{m}}_{-}$ due to finite-size fluctuations, similarly to what happens in a bistable system. As the temperature is increased beyond $T_{c}$, there is a transition to an unimodal regime in which *m* oscillates around zero, where the amplitude of oscillations decreases with *T* and eventually vanishes in the T≫1 limit that corresponds to pure noise. The transition at $T_{c}$ becomes more abrupt as the period τ of the field increases. We also studied the response of the system to the external field by means of the distribution of residence times, i.e., the time interval between two consecutive changes of the sign of *m*. We found that there is an optimal temperature $T^{*}$ for which the response is maximum, that is, a stochastic resonance phenomenon induced by the external noise controlled by *T*. Furthermore, we developed an MF approach that lead to a non-linear rate equation for the time evolution of *m* in the thermodynamic limit, whose numerical solution agrees very well with MC simulations of the model. We used this equation to give a numerical estimate of $T_{c}$, and found that the behavior of $T_{c}$ with the period τ is qualitatively similar to that of $T^{*}$. Although the transition temperature $T_{c}$ is similar to the resonance temperature $T^{*}$ only for large τ, this analysis shows that they are related.

A possible interpretation of these results in a social context is the following. Reacting with a contrarian attitude occasionally (small *T*/low noise) on a given issue, that is, adopting an opposite position to that of the majority of our acquaintances, leads to a state of collective agreement in a population, which can be reversed completely after some time by means of a collective decision, independently of the external propaganda. This alternating behavior between opposite opinions might be seen as more “socially healthy” than a frozen full consensus in one of the two alternatives, which happens in populations with a total absence of contrarian attitudes (T=0). However, having a contrarian behavior more often could induce a collective state where the mean opinion oscillates in time following the external propaganda, which can be interpreted as a society whose opinions are manipulated optimally by the mass media, in opposition to collective freedom. Finally, in the extreme case of having a very frequent contrarian attitude (T≫1), the population falls into a state of opinion bipolarization, where there are two groups of similar size with opposite opinions.

Given that the opinion formation model introduced and studied in this article is simple enough, it allowed for a mathematical treatment that led to numerical and analytical results. However, due to its simplicity, the model is not very adequate to describe the complexity observed in real social systems, and, thus, some extensions are necessary to cope with real life scenarios. For instance, one can possibly collect real data to infer how mass media propaganda evolves over time. We could expect that real propaganda oscillates with a frequency that is not constant like in the model, but varies in time, and is centered at an arbitrary positive or negative number in the interval (−1,1). Nevertheless, we have seen that the model provides insights on features that could be actually relevant in the real word, like the conditions for which the opinions of a society are optimally manipulated by mass media, as described in the paragraph above, related to the stochastic resonance effect.

The results presented in this article correspond to a fully connected network. Although we expect that the conclusions remain valid qualitatively for other interaction topologies, it might be worthwhile to study the model in complex networks like scale-free or Erdös Renyi networks, which better-represent social interactions. This version of the model would be more realistic, not only for the interaction topology, but also because the size of the majority group would be different for each agent or node in the network (its degree or number of neighbors), allowing for the case of ties if the group has an even number of agents. It might also be interesting to explore how the stochastic resonance effect depends on the topology of the network. Finally, the model can be easily adapted to include some of the possible real time features of mass media propaganda described above. We leave all these ideas for future work.

## Data Availability

All relevant data are within the paper.

## Glossary

Symbol	Name
$s_{i}=\pm 1$	Opinion of agent *i*;
*N*	Number of agents;
*t*	Time;
Δt=1/N	Time step;
p(t)	Majority probability;
H(t)	External field;
$H_{0}$	Amplitude of H(t);
τ	Period of H(t);
*T*	Temperature;
$T^{*}$	Resonance temperature;
$T_{c}$	Transition temperature;
$\sigma_{\pm }$	Fraction of agents with ±1 opinion;
*m*	Magnetization;
〈|m|〉	Ensemble average of absolute value of *m*;
$\bar{m}$	Time average of *m*;
$t_{r}$	Residence time;
RTD	Residence time distribution;
A	Response;
GMM	Galam majority rule;
VM	Voter Model;
MF	Mean field;
SR	Stochastic Resonance.
